# Microglial motility in Alzheimer’s disease and after Aβ42 immunotherapy: a human *post-mortem* study

**DOI:** 10.1186/s40478-019-0828-x

**Published:** 2019-11-08

**Authors:** Diana K. Franco-Bocanegra, Bethany George, Laurie C. Lau, Clive Holmes, James A. R. Nicoll, Delphine Boche

**Affiliations:** 1 Clinical Neurosciences, Clinical and Experimental Sciences Academic Unit, Faculty of Medicine, University of Southampton, Southampton General Hospital, Mailpoint 806, Southampton, SO16 6YD UK; 2 Clinical and Experimental Sciences, Faculty of Medicine, Sir Henry Wellcome Laboratories, University of Southampton, Southampton, UK; 3 Memory Assessment and Research Centre, Moorgreen Hospital, Southern Health Foundation Trust, Southampton, UK; 4 Department of Cellular Pathology, University Hospital Southampton NHS Foundation Trust, Southampton, UK

**Keywords:** Human microglia, Cell motility, Alzheimer’s disease, Aβ-immunotherapy, Neuroinflammation

## Abstract

Microglial function is highly dependent on cell motility, with baseline motility required for homeostatic surveillance activity and directed motility to migrate towards a source of injury. Experimental evidence suggests impaired microglial motility in Alzheimer’s disease (AD) and therefore we have investigated whether the expression of proteins associated with motility is altered in AD and affected by the Aβ immunotherapy using *post-mortem* brain tissue of 32 controls, 44 AD cases, and 16 AD cases from our unique group of patients immunised against Aβ42 (iAD).

Sections of brain were immunolabelled and quantified for (i) the motility-related microglial proteins Iba1, cofilin 1 (CFL1), coronin-1a (CORO1A) and P2RY12, and (ii) pan-Aβ, Aβ42 and phosphorylated tau (ptau). The neuroinflammatory environment was characterised using Meso Scale Discovery multiplex assays. The expression of all four motility-related proteins was unmodified in AD compared with controls, whereas Iba1 and P2RY12, the homeostatic markers, were increased in the iAD group compared with AD. Iba1 and P2RY12 showed significant positive correlations with Aβ in controls but not in the AD or iAD groups. Pro- and anti-inflammatory proteins were increased in AD, whereas immunotherapy appears to result in a slightly less pro-inflammatory environment.

Our findings suggest that as Aβ appears during the ageing process, the homeostatic Iba1 and P2RY12 –positive microglia respond to Aβ, but this response is absent in AD. Aβ-immunisation promoted increased Iba1 and P2RY12 expression, likely reflecting increased baseline microglial motility but without restoring the profile observed in controls.

## Introduction

Evidence from genetics, experimental and human *post-mortem* studies [47] supports the involvement of microglia, the resident immune cells of the brain, in the pathological cascade that leads to the development of Alzheimer’s disease (AD). However, their role in the disease is still not fully understood [31]. In the healthy brain, microglia survey the parenchyma, constituting its first line of defence against pathogens or injury [10, 34], and interact with neurons helping to shape synaptic communication [29, 52–54, 56].

One of the most prominent characteristics of microglia is their motility, which is essential for the cells to perform their functions. Two types of motility are present in microglial cells: *baseline motility*, which consists of the extension, retraction, and movement of the microglial processes [18, 37], allowing microglia to survey their environment, clear cellular debris, interact with neurons and synapses, and remodel the extracellular matrix [34]. If, while performing this baseline motility, a microglial cell encounters molecules that could be indicative of infection or tissue damage, the cell will then switch to *directed motility*. Directed motility consists of a targeted extension of processes towards the source of injury [18]. At the molecular level, microglial motility depends on two mechanisms: i) reorganisation of the actin cytoskeleton and ii) purinergic signalling [13].

The actin cytoskeleton consists of a dynamic filament network, regulated by a complex machinery that involves several proteins. Some of the most important actin-related proteins in the microglial cytoskeleton are the coronins, the cofilins and the ionised calcium-binding adapter molecule (Iba1) [1, 46]. The coronin gene family contains six members with roles as regulators of actin dependent processes, such as cell motility and vesicle trafficking [40, 41]. Coronins localise to sites of dynamic actin assembly, regulating filament organisation via interactions between actin and the Arp2/3 protein complex. This results in the building of branches in the actin filament network [19]. Coronin 1a (CORO1A) plays a large role in the normal immune system as the most prominent coronin in cells of haematopoietic origin [12], and has been previously identified in human microglia [1]. Cofilins, and particularly cofilin 1 (CFL1), are actin-binding proteins that depolymerise and separate actin filaments [44]. Lastly, Iba1 is a cross-linking protein, the role of which is to organise actin filaments into networks. In microglia, Iba1 is crucial for the formation of actin bundles, [35], an event necessary for the construction of lamellipodia, filopodia, and membrane ruffles. These structures are essential for microglial migration and phagocytosis. Due to its relatively homogeneous distribution along the cell body and processes, Iba1 has been widely used as a marker to identify microglia in humans and experimental models. Purinergic signalling plays an important role in microglial motility. The presence of extracellular nucleosides (ATP, ADP, UTP, UDP), released as a result of neuronal death, triggers the activation of P2Y receptors. The ADP receptor P2RY12 is now regarded as a microglia-specific marker, mainly associated with directed motility, driving chemotaxis through a mechanism that involves the activation of potassium (K^+^) channels [26]. Therefore, both actin reorganisation and purinergic signalling are essential to the two forms of microglial motility.

Alterations of microglial motility have been documented in AD, with (i) microglial clusters identified around Aβ plaques indicating movement of microglia as part of their response to Aβ aggregation [16, 57], (ii) in AD mouse models, impairment of directed motility and phagocytic activity [23], and (iii) morphological changes in plaque-associated cells [38]. Aβ-immunotherapy is one of the major current therapeutic approaches being investigated in AD [51]. The first clinical trial of Aβ immunotherapy in humans involved active immunisation with a full-length synthetic Aβ42 compound named AN1792 [3]. Our neuropathological study of these patients has shown a markedly reduced plaque load associated with microglial changes [57, 58]. However, these changes were not associated with reduced cognitive decline [17, 33].

In this study, we have explored whether the expression of proteins associated with motility is altered in AD and affected by the Aβ immunotherapy using *post-mortem* brain tissue of controls, AD cases, and our unique group of AD cases immunised against Aβ42.

## Material and methods

### Cases

Brain tissue from 76 donors was sourced from the South West Dementia Brain Bank comprising 44 AD cases and 32 controls. Alzheimer’s cases had a clinical diagnosis of sporadic AD made during life and satisfied *post-mortem* neuropathological consensus criteria for AD [20]. Cases with any other significant brain pathologies such as stroke, tumour, or traumatic brain injury were excluded from the study. Controls were aged-matched cases, with no history of neurological or psychiatric disease or symptoms of cognitive impairment. Additionally, 16 immunised AD subjects (iAD) who participated in the AN1792 clinical trial were also included in the study. The characteristics of the groups are presented in Table 1 and individual details in the Additional file 1: Tables S1, S2 and S3.

**Table 1:** Demographic, clinical and *post-mortem* characteristics of the three groups

Groups		Control *n* = 32	AD *n* = 44	iAD *n* = 16
Gender		17F:15M	28F:16M	7F:9M
Age of Death (years, mean ± SD)		84 ± 7	80 ± 6	79 ± 8
Age of AD onset (years, mean ± SD)		n/a	70 ± 7	67 ± 8
Duration of AD (years, mean ± SD)		n/a	10 ± 3	12 ± 4
Braak Stage		0-II: 29 III-IV: 3 V-VI: 0	0-II: 0 III-IV: 4 V-VI: 40	0-II: 0 III-IV: 1 V-VI: 15
*APOE* genotype	*ε4/−*	3/28 (10.7%)	13/38 (34.2%)	6/10 (60.0%)
	*ε4/ε4*	1/28 (3.6%)	9/38 (23.7%)	3/10 (30.0%)
*Post-mortem* delay (hours, mean ± SD)		42 ± 23	42 ± 26	22 ± 25

*Control* neurologically/cognitively normal controls, *AD* Alzheimer’s disease cases, *iAD* immunised Alzheimer’s disease cases

*F* female, *M* male,

*APOE* genotyping was not available for all cases

*n/a* not-applicable, *SD* standard deviation

Cerebral cortex from the inferior parietal lobule was investigated in all cases. This is one of the areas recommended for neuropathological assessment by the CERAD (Consortium to Establish a registry for Alzheimer’s Disease) [11] as it is significantly affected by AD pathology. Formalin-fixed paraffin embedded tissue was used for the immunodetection of neuropathological and neuroinflammatory markers. Frozen tissue, available for 31 controls, 35 AD cases and 11 iAD cases was used for detection of inflammation-related proteins by Meso Scale Discovery multiplex assays.

### Immunohistochemistry

Immunohistochemistry was performed on 4 μm paraffin-embedded sections, targeting the markers of AD pathology pan-Aβ (clone 4G8, BioLegend), Aβ42 (clone 21F12, Elan Pharmaceuticals Ltd) and the phosphorylated (p) tau (clone AT8, ThermoScientific) protein, as well as the microglial motility-related proteins Iba1 (rabbit polyclonal, Wako Chemicals), CFL1 (polyclonal rabbit, ThermoScientific), CORO1A (rabbit polyclonal, LifeSpan Biosciences) and P2RY12 (rabbit polyclonal, Sigma Aldrich). Bound antibodies were visualized using the avidin–biotin–peroxidase complex method (Vectastain Elite, Vector Laboratories) with 3,3′-diaminobenzidine as chromogen and 0.05% hydrogen peroxide as substrate (Vector Laboratories). All sections were counterstained with haematoxylin, then dehydrated and mounted in Pertex (Histolab Products AB). The staining was performed in two batches with each batch containing cases from all groups (Control, AD, iAD). All experiments included a negative control slide incubated in buffer with no primary antibody and a positive control slide containing a specific tissue type known to express the protein of interest (e.g. tonsil).

### Quantification of staining

Quantification was blinded to the case designation and performed on the grey matter in the same sulcus of the inferior parietal lobule for all cases. For each antibody, the slides were scanned together at a magnification of × 20 in an automated slide scanner microscope Olympus VS110 (Olympus America Inc.) and visualised with Olympus VS-Desktop software using a specialised add-in that allows the extraction of the region of interest (ROI) of 0.25mm^2^. For each slide, 30 ROIs were selected in a zig-zag pattern within the grey matter, ensuring coverage of all cortical layers. Extracted ROIs were analysed with the ImageJ software (version 1.49u, Wayne Rasband, NIH, USA) using an automated macro specific to each antibody. The area fraction labelled by the antibody in each ROI was obtained by quantifying the presence or absence of the staining in each pixel, expressed as protein load (%).

### Cytokine assay

Inflammatory proteins were measured on the V-Plex Meso Scale Discovery (MSD) electrochemiluminescence multi-spot assay platform (MesoScale Diagnostics, Rockville USA). 100 mg of frozen grey matter were homogenised at a tissue concentration of 20% w/v in RIPA lysis buffer (Thermo Fisher Scientific), supplemented with protease inhibitors (Sigma Aldrich) and phosphatase inhibitors (Thermo Fisher Scientific). Total protein concentration in the supernatant was measured by BCA Protein Assay Kit (Thermo Fisher Scientific). 25 μl of brain homogenate (1:2 dilution) were used for the V-PLEX human proinflammatory panel 1 and cytokine panel 1, and each plate was read on a Meso Quickplex SQ120 (Meso Scale Discovery) according to manufacturer’s instructions. Absolute target protein levels (pg/ml) were obtained and normalised with respect to the total protein concentration, as obtained by the BCA assay. Three frozen homogenates from a multiple sclerosis brain containing either chronic inactive, acute or chronic active lesions were included as positive controls.

### Statistical analysis

Statistical analysis was performed using the IBM SPSS v24 statistical software package (SPSS Inc. Chicago IL). The normality of distribution of each marker across the groups was assessed by the Shapiro-Wilk test. Data were not normally distributed, and thus non-parametric tests were used. Comparisons among study groups were performed using the Kruskal-Wallis test. The Spearman’s test was performed to assess correlations between variables – to explore the relationship (i) between microglial motility proteins and key features of AD pathology (Aβ and tau loads), and (ii) between the microglial-motility proteins. To account for multiple testing, the Benjamini-Hochberg procedure to control for the false discovery rate (FDR) was used as *post-hoc* correction. For all tests, an adjusted *P* value < 0.05 was considered significant.

## Results

### Aβ and tau pathology

**Fig. 1:**
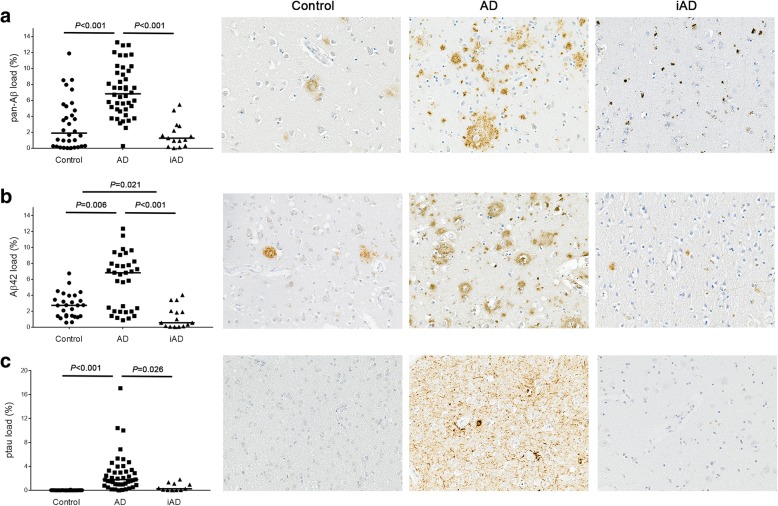
Illustration and quantification of pan-Aβ, Aβ42 and ptau loads for the 3 groups. **a** We observe a significantly increased pan-Aβ load in AD vs. controls (*P* < 0.001), whereas it is significantly decreased in iAD vs. AD (*P* < 0.001). **b** Aβ42 quantification shows similar expression than pan-Aβ, with also a significantly decreased load in iAD vs. control groups (*P* = 0.021). **c** Phosphorylated (p) tau shows a significantly increased load in AD vs. control (*P* < 0.001) while it is decreased in iAD vs. AD groups (*P* = 0.026). Counterstaining: Haematoxylin. Scale bar = 50 μm

Aβ and ptau immunoreactivity was quantified in order to characterise the cases with respect to AD pathology and to permit correlations with the microglial motility-related proteins. A significantly increased pan-Aβ load was observed in AD compared to control cases (*P* < 0.001), whereas it was significantly decreased in the iAD group compared to AD cases (*P* < 0.001), as expected. No significant difference was observed between control and iAD cases (Fig. 1a). Aβ42 load was significantly increased in AD vs control cases (*P* = 0.006) and decreased in iAD vs AD cases (*P* < 0.001), as expected. Interestingly, Aβ42 load, was also significantly decreased in iAD compared to control cases (*P* = 0.021) (Fig. 1b). Ptau was significantly increased in AD vs control cases (*P* < 0.001) and decreased in iAD vs AD cases (*P* = 0.026), with no difference between the iAD and control groups (Fig. 1c).

### Microglial motility-related proteins

**Fig. 2:**
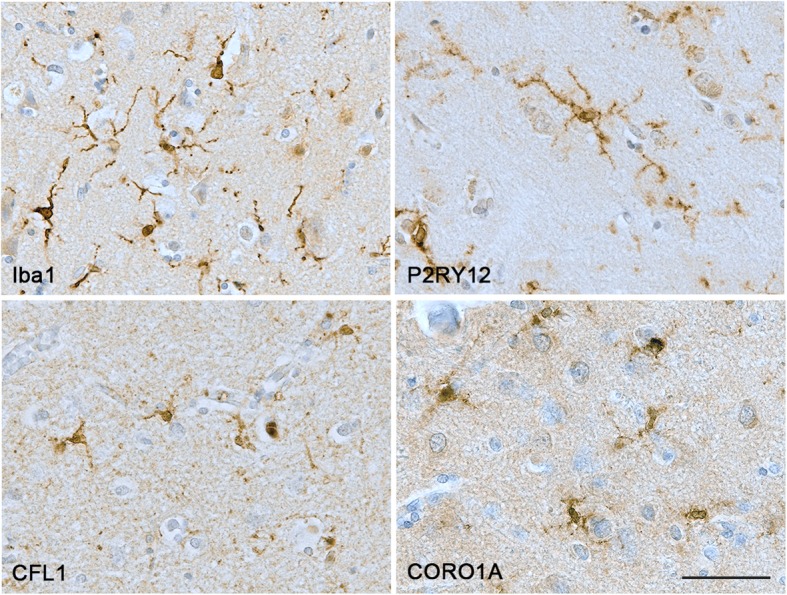
Illustration of the immunostaining obtained with the different microglial motility-related proteins. Counterstaining: Haematoxylin. Scale bar = 50 μm

Immunohistochemistry for the four proteins related to microglial motility was investigated in the cortical grey matter. Iba1, CFL1 and CORO1A immunolabelled microglia and perivascular macrophages and in AD microglial clusters around amyloid plaques. While P2RY12 was specific to microglia with no labelling of perivascular macrophages (Fig. 2).

**Fig. 3:**
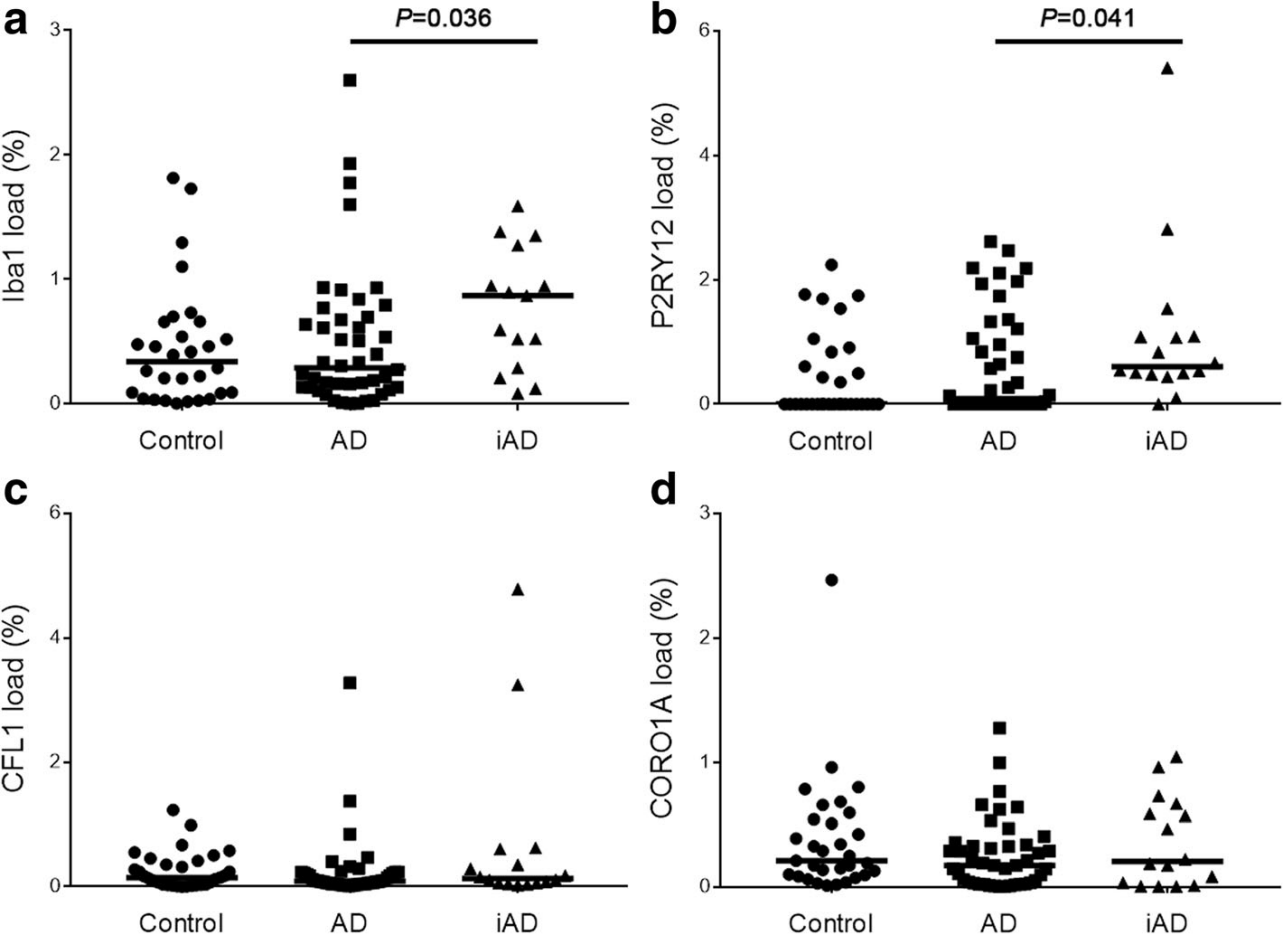
Quantification of the microglial motility-related proteins in the three groups. No difference was observed between the AD and control groups for the four proteins (**a**-**d**). Significantly increased Iba1 and P2RY12 loads were detected in the iAD vs. AD groups only (*P* = 0.036 and *P* = 0.041, respectively) (**a**-**b**)

Quantification of these four proteins showed no significant differences between AD and the control group: Iba1: control = 0.34% (IQR: 0.08–0.63%) vs AD = 0.29%(IQR: 0.13–0.68%), *P* = 0.788; CFL1: control = 0.15% (IQR: 0.03–0.038%) vs AD = 0.08% (IQR: 0.04–0.022%), *P* = 0.314; P2RY12: control = 0.00% (IQR: 0.00–0.55%) vs AD = 0.09% (IQR: 0.00–1.09%), *P* = 0.290; CORO1A: control = 0.21% (IQR: 0.10–0.52%) vs AD = 0.17% (IQR: 0.03–0.33%), *P* = 0.406. However, there was a significant increase for Iba1 and P2RY12 in iAD compared to AD cases: Iba1: AD = 0.29% (IQR: 0.13–0.68%) vs iAD = 0.87% (IQR: 0.40–1.11%), *P* = 0.036; P2RY12: AD = 0.09% (IQR: 0.00–1.09%) vs iAD = 0.60% (IQR: 0.50–1.08%), *P* = 0.041 (Fig. 3). No significant difference was found in CFL1 and CORO1A loads between the AD and iAD groups: CFL1: AD = 0.08% (IQR: 0.04–0.022%) vs iAD 0.13% (IQR: 0.06–0.41%), *P* = 0.314; CORO1A: AD = 0.17% (IQR: 0.03–0.33%) vs iAD = 0.20% (IQR: 0.03–0.61%), *P* = 0.406).

We then explored whether the expression of the microglial motility-related proteins was correlated with the proteins that aggregate in AD. In the control group, correlations were detected for Iba1 and P2RY12 with (i) pan-Aβ (r_s_ = 0.440, *P* = 0.046 and r_s_ = 0.443, *P* = 0.042; respectively) and (ii) Aβ42 (r_s_ = 0.691, *P* < 0.001 and r_s_ = 0.629, *P* < 0.001; respectively) (Table 2). No correlations were observed with pan-Aβ and Aβ42 in the AD or iAD groups, and with ptau for the three groups.

**Table 2:** Correlation of motility-related markers with pan-Aβ, Aβ42 and ptau

		Iba1	CFL1	P2RY12	CORO1A
pan-Aβ	Control	*r*_*s*_ = 0.440*	r_s_ = 0.301	*r*_*s*_ = 0.443*	r_s_ = 0.055
	AD	r_s_ = −0.055	r_s_ = 0.077	r_s_ = 0.219	r_s_ = 0.009
	iAD	r_s_ = −0.143	r_s_ = 0.011	r_s_ = 0.054	r_s_ = −0.029
Aβ42	Control	*r*_*s*_ = 0.691***	r_s_ = 0.459	*r*_*s*_ = 0.629***	r_s_ = 0.051
	AD	r_s_ = −0.021	r_s_ = − 0.172	r_s_ = 0.078	r_s_ = − 0.158
	iAD	r_s_ = −0.464	r_s_ = −0.575	r_s_ = 0.171	r_s_ = 0.464
ptau	Control	rs = −0.113	rs = −0.165	rs = 0.017	rs = 0.-173
	AD	rs = −0.239	rs = −0.043	rs = − 0.249	rs = − 0.019
	iAD	rs = −0.709	rs = − 0.118	rs = 0.191	rs = 0.036

*r*_*s*_ Spearman’s rank correlation, *P < 0.05; *** *P* < 0.001, significant *P* values are in italic

*CFL1* cofilin 1, *CORO1A* coronin-1A

*Control* neurologically/cognitively normal controls, *AD* Alzheimer’s disease cases, *iAD* immunised Alzheimer’s disease cases, *ptau*: phosphorylated tau

We also assessed whether the different mechanisms involved in motility (actin-related or purinergic signalling) were related. In the control group, Iba1 was correlated with CFL1 (r_s_ = 0.502, *P* = 0.025) and P2RY12 (r_s_ = 0.572, *P* = 0.005). In the AD cases, Iba1 was related to CFL1 (r_s_ = 0.559, *P* < 0.001), CORO1A (r_s_ = 0.436, *P* = 0.014) and P2RY12 (r_s_ = 0.558, *P* < 0.001). CFL1 was also correlated with CORO1A (r_s_ = 0.530, *P* = 0.002) and P2RY12 (r_s_ = 0.542, *P* = 0.001), and CORO1A with P2RY12 (r_s_ = 0.357; *P* = 0.049). No correlation was detected in the iAD group (Table 3).

**Table 3:** Correlations between the motility-related markers

	Control	AD	iAD
Iba1 vs. CFL1	*r*_*s*_ = 0.502**	*r*_*s*_ = 0.559***	r_s_ = 0.443
Iba1 vs. CORO1A	r_s_ = 0.357	*r*_*s*_ = 0.436**	r_s_ = 0.361
Iba1 vs. P2RY12	*r*_*s*_ = 0.572**	*r*_*s*_ = 0.558***	r_s_ = 0.114
CFL1 vs. CORO1A	r_s_ = 0.376	*r*_*s*_ = 0.530**	r_s_ = 0.412
CFL1 vs. P2RY12	r_s_ = 0.223	*r*_*s*_ = 0.542***	r_s_ = 0.068
CORO1A vs. P2RY12	*r*_*s*_ = 0.128	*r*_*s*_ = 0.357*	r_s_ = 0.232

*r*_*s*_ Spearman’s rank correlation; *P < 0.05; **P < 0.01; ***P < 0.001; significant *P* values are in italic

*CFL1* cofilin 1, *CORO1A* coronin-1A

*Control* neurologically/cognitively normal controls, *AD* Alzheimer’s disease cases, *iAD* immunised Alzheimer’s disease cases

### Neuroinflammatory environment

To explore the relationship between microglial motility and the neuroinflammatory environment, the MSD platform was used to measure the levels of IFNγ, IL1β, IL2, IL4, IL6, IL8, IL10, IL12p70, IL13, TNFα, IL1α, IL5, IL7, IL12/IL23p40, IL15, IL16, IL17A, GM-CSF, TNFβ and VEGF in the three groups. Significant increases in the AD group compared to the control group were observed for: IFNγ (*P* < 0.001), IL10 (*P* = 0.003), IL12p70 (*P* < 0.001), IL13 (*P* < 0.001), IL2 (*P* = 0.002), IL4 (*P* = 0.007), TNFα (*P* < 0.001), IL15 (*P* = 0.024), IL16 (*P* = 0.020) and TNFβ (*P* = 0.009). In the iAD group compared to AD, IL13 (*P* = 0.017), IL8 (*P* = 0.023) and VEGF (*P* < 0.001) were significantly increased; whereas IL7 was significantly decreased (*P* = 0.012). Of note, of the proteins that were significantly different between AD and iAD, IL13 and VEGF were also significantly increased with respect to the control levels (IL13: *P* < 0.001; VEGF: *P* = 0.012), while IL7 was significantly decreased (*P* = 0.003) (Table 4).

**Table 4:** Comparison of levels of inflammation-related proteins measured by V-PLEX Meso Scale Discovery Multiplex Assays

pg/μg total protein	Control	AD	iAD	*P* value^1^	*P* value^2^
IFNγ	0.125 (0.085-0.206)	0.286 (0.147-0.377)	0.139 (0.052-0.313)	*<0.001 (2.3)*	0.167
IL1β	0.195 (0.097-0.274)	0.125 (0.085-0.257)	0.208 (0.167-0.427)	0.175	0.175
IL10	0.024 (0.016-0.040)	0.047 (0.033-0.055)	0.038 (0.026-0.050)	*0.003 (1.9)*	0.301
IL12p70	0.204 (0.156-0.255)	0.367 (0.280-0.478)	0.364 (0.317-0.424)	*<0.001 (1.8)*	0.986
IL13	0.566 (0.424-0.742)	0.985 (0.786-1.145)	1.718 (1.199-1.870)*	*<0.001 (1.7)*	*0.017 (1.7)*
IL2	0.084 (0.067-0.120)	0.141 (0.113-0.180)	0.173 (0.143-0.222)	*0.002 (1.7)*	0.172
IL4	0.023 (0.017-0.033)	0.045 (0.038-0.056)	0.042 (0.035-0.051)	*0.007 (1.9)*	0.779
IL6	0.644 (0.192-1.015)	0.347 (0.209-0.573)	0.585 (0.187-1.445)	0.482	0.482
IL8	2.694 (1.428-6.597)	1.530 (1.221-3.419)	2.796 (2.300-5.561)	0.081	*0.023 (1.8)*
TNFα	0.112 (0.089-0.141)	0.200 (0.147-0.247)	0.196 (0.152-0.215)	*<0.001 (1.8)*	0.575
GM-CSF	0.000 (0.000-0.001)	0.000 (0.000-0.002)	0.000 (0.000-0.000)	0.682	0.682
IL1α	0.043 (0.026-0.065)	0.047 (0.029-0.082	0.044 (0.034-0.079)	0.735	0.735
IL12/IL23p40	0.079 (0.054-0.116)	0.064 (0.044-0.100)	0.094 (0.072-0.132)	0.163	0.163
IL15	0.637 (0.493-0.711)	0.744 (0.638-0.929)	1.026 (0.768-1.366)	*0.024 (1.2)*	0.053
IL16	19.30 (14.33-24.84)	30.19 (20.65-46.03)	35.13 (25.41-46.57)	*0.020 (1.6)*	0.617
IL17A	0.000 (0.000-0.000)	0.000 (0.000-0.000)	0.000 (0.000-0.000)	0.839	0.839
IL5	0.000 (0.000-0.000)	0.000 (0.000-0.000)	0.000 (0.000-0.000)	0.677	0.677
IL7	0.036 (0.022-0.049)	0.027 (0.013-0.064)	0.003 (0.000-0.015)*	0.323	*0.012 (0.1)*
TNFβ	0.005 (0.003-0.007)	0.012 (0.004-0.016)	0.005 (0.000-0.013)	*0.009 (2.4)*	0.143
VEGF	1.630 (0.937-4.511)	1.056 (0.537-2.436)	5.524 (4.292-6.497)*	0.066	*<0.001 (5.2)*

Values are median with IQR, *P* value by Kruskal-Wallis test with Dunn’s test as *post hoc* pairwise comparison;

Significant *P* value in italic with the fold change between brackets

^1^AD *vs*. control, ^2^iAD *vs*. AD

*Control* neurologically/cognitively normal controls, *AD* Alzheimer’s disease cases, *iAD* immunised Alzheimer’s disease cases

* These values are significantly different from both AD and the control group (IL13: *P*<0.001 (3.03); IL7: *P*=0.003 (0.08); VEGF: *P*=0.012 (-3.39)

## Discussion

Evidence from animal studies shows that microglia are highly motile cells [14, 16, 34, 49], with motility impaired in AD models [6, 23]. This highlights motility as a functional aspect of microglia relevant to disease pathogenesis. Our aim was to characterise the expression of motility-related microglial proteins in the human brain and to evaluate its changes in AD and after Aβ immunotherapy. In the present study, we observed increased expression of the microglial homeostatic markers Iba1 and P2RY12 in the immunised group, which was associated with a less pro-inflammatory environment than observed in AD. We acknowledge that this approach has limitations, partly inherent to any *post-mortem* study, which reside in the absence of exploration of the temporal relationship between the different markers investigated, and with the analysis limited to the late-stage consequences of AD. Additionally, other proteins such as actin, the Arp2/3 complex, or the integrins also relevant for motility have not been evaluated here, as our investigation focuses on microglial-specific motility-related proteins. The strength of the study is in the examination of AD itself, rather than an experimental model of the disease which does not represent the whole extent of the complexity of the human disease. Specifically, most rodent models lack tau pathology, the generation and propagation of which microglia are proposed to have an important role. The novelty of our study resides in the combined quantitative assessment of several markers associated with microglial motility in relation to the neuroinflammatory environment, and the accumulation of Aβ and ptau. This study also takes advantage of the exceptional chance to explore the effect of Aβ42-immunotherapy which constitutes an important topic of study since there is currently substantial resource being invested in this therapeutic approach.

### Aβ and tau pathology

As expected, the expression of the key features of AD neuropathology, Aβ and ptau, was increased in AD. The effect of the immunotherapy on AD has been previously reported by us [4, 32, 33, 42]. However in this study, we were able to compare the effects of the treatment with both controls and unimmunised AD. Interestingly, we observed that Aβ42 immunotherapy reduced the overall Aβ levels to those of controls, indicating an effect not restricted to the Aβ42 form. This is consistent with our studies on the same immunised cohort using antibodies specific for the Aβ40, Aβ42 and Aβ43 species and reporting significant decrease of the three species [21, 32].

Of note, its effect on the Aβ42 form seems to be more pronounced, with a reduction to significantly lower levels than those of controls. This highlights a possible higher affinity of the immunisation towards the Aβ42 species, perhaps due to the fact that full-length Aβ42 was the immunogen originally used in the AN1792 trial, even though earlier studies showed that the antibodies produced by the patients were primarily N-terminal specific [24]. This implies that the antibodies are able to target all different species of Aβ. The ptau load was reduced to the level of the controls, confirming the effect of Aβ-immunotherapy on the tau pathology [4, 33]. Indeed, our previous studies on the immunised cohort showed that ptau load was reduced after Aβ immunisation, in association with a lower expression of the tau phosphorylating enzyme GSK-3b [2]. Ptau load is mainly constituted of tau-containing dystrophic neurite clusters and ptau in neuronal processes [4], rather than tangles. Nevertheless, despite the decrease in ptau load as assessed by quantification in specific localised neuroanatomical regions of cortex, most of the iAD subjects still had progressed to Braak stages V or VI, as evaluated by the anatomical distribution of neurofibrillary tangles throughout the brain areas. We interpret these findings as indicating that localised removal of Aβ following Aβ immunotherapy results in a corresponding removal of ptau in that location, consistent with the amyloid cascade hypothesis. However, it does not stop the spread of ptau through the brain, consistent with the evidence for prion-like spread of ptau [4, 33].

### Microglial motility-related proteins

In AD, expression of all four motility-related proteins examined was not modified compared with controls, consistent with another *post-mortem* AD study [45]. In this paper, they evaluated expression of Iba1 and P2RY12 and also found no difference in the expression of both proteins between the control and AD groups. Interestingly, in that study, when the AD cases were split according to the Braak stage, a decrease for both Iba1 and P2RY12 proteins was observed in the late-stage of the disease (Braak stage V/VI) but only in the hippocampal area, a region heavily affected by neuronal loss and tau accumulation [50]. In our study, even though most of the AD cases correspond to Braak stages V and VI, we did not observe decrease in these proteins, which could be indicative of regional differences between the hippocampus and the cerebral cortex due to the exacerbated degree of neuronal death and tau aggregation in the hippocampal region in late stages of the disease. With respect to CFL1, our observation confirms previous reports of no difference in CFL1 expression in AD [39]. The presence of CORO1A has been observed in human microglia [1], but to our knowledge has never been assessed in AD.

After Aβ immunotherapy in AD, a 3-fold increase in Iba1 expression and a 6.6-fold increase in P2RY12 expression was observed. Data on anti-Aβ antibody titres measured in the blood during life some years earlier were available [57] and did not correlate with the microglial analyses. Iba1 is expressed by microglia in both physiological and disease conditions, suggesting that Iba1 participates in both baseline and directed motility. In vitro studies have characterised the role of Iba1 in relation to membrane ruffling, and the formation of phagocytic cups is Iba1-dependent [35]. Therefore, the higher Iba1 expression observed is in accordance with the reported increased phagocytic activity associated with Aβ clearance [58] as the consequence of immunotherapy stimulating microglial phagocytosis towards Aβ. Thus, after immunotherapy, Iba1 expression is likely to reflect the motility changes associated with phagocytosis. Similarly to Iba1, P2RY12 is detected on all microglial cells and a role of P2RY12 in directed motility is also well-documented. The release of ATP to the extracellular space, caused by cell death, and its subsequent hydrolysis to ADP activates P2RY12 [48]. Studies have shown that microglia from mice lacking P2RY12 expression show normal baseline motility but impaired directed motility [15, 26]. Moreover, it was observed that P2RY12 expression in ramified microglia was markedly decreased as cells turn to the reactive/amoeboid form [15, 38]. This suggests a role of P2RY12 in directed motility taking place at an early stage of the cell activation. Thus, P2RY12 expression after immunotherapy might suggest an increase both in baseline and directed motility. This is also consistent with the neuroinflammatory environment observed in the immunised group, which is slightly leaning towards a more anti-inflammatory profile, considering evidence that P2RY12 is downregulated under pro-inflammatory conditions and upregulated in an anti-inflammatory context [22, 30]. Of note, a previous study by our group in the immunised group showed that neuronal loss is correlated with Iba1-positive microglia and phagocytic CD68-positive microglia associated with pro-apoptotic neurons, but without accelerated cognitive decline compared to non-immunised AD patients [36]. This suggests that immunotherapy may enhance microglial elimination of damaged and dysfunctional neurons. Considering the role of P2RY12 as a purinergic receptor upregulated with the release of ATP that occurs during cell damage, it could be hypothesized that the increased P2RY12 expression in the immunised cohort reflects microglial increased response, as a result of the treatment, towards damaged neurons.

### Motility-related proteins and the pathology

Relationships between the microglial motility-related proteins and Aβ and ptau, key pathological features of AD, were explored. In controls, Iba1 and P2RY12 were associated with Aβ, with these associations lost in AD and not restored by the immunotherapy. It is recognised that with ageing, Aβ accumulates independently of the dementia status [8]. Therefore, the relationship of Iba1 and P2RY12 with Aβ in the absence of dementia may reflect a healthy chemotactic microglial response towards Aβ, via the directed motility mechanism, as previously reported in the Cognitive Function and Ageing Studies group [28]. In AD, these correlations were lost, possibly due to either (i) Aβ over-accumulation to a level at which microglia are overwhelmed and no longer capable of responding accordingly, (ii) microglia attracted to plaques and been immobilised there [57], or (iii) microglia being “distracted” by the tau pathology [28]. However, the absence of correlation of the motility-related proteins with Aβ or ptau in the AD group is in favour of a microglial response towards other pathological changes, such as the synaptic/neuronal loss. Of note, the correlations were not restored after immunotherapy despite Aβ levels as lower as in the controls and this despite the increased Iba1 and P2RY12 expression. No relationship was observed with CFL1 or CORO1A.

Since no correlations were found between the microglial motility-related markers and ptau, this implies that the motility response driven by the proteins studied is independent of tau, or at least they do not directly interact with tau.

### Interactions between motility-related proteins

Correlations between the microglial motility-related proteins were also investigated. In controls, Iba1 correlated with CFL1 and P2RY12, reflecting an interaction between the actin-related motility mechanisms and the purinergic signalling pathway in physiological conditions. Interestingly, in AD all markers were related to each other, implying that the pathogenesis elicited a coordinated response of the microglial motility proteins as part of the pathogenesis spreading. In the immunised group, no relation was observed despite the increased expression of the homeostatic markers Iba1 and P2RY12. This implies that immunotherapy did not restore the physiological profile of microglial protein interactions.

### Neuroinflammatory environment

Inflammation-related proteins were evaluated to characterise the microenvironment. In AD, 10 were increased, including the pro-inflammatory proteins IFNγ, TNFα, TNFβ, IL12p70, IL2, IL15 and IL16, and the anti-inflammatory proteins IL4, IL10 and IL13. This reflects that both pathways of activation coexist in human AD as previously reported [9, 55].

After Aβ immunotherapy, only four markers were modified when compared with unimmunised AD, with increased expression of the pro-inflammatory chemokine IL8, the anti-inflammatory cytokine IL13, the vascular endothelial growth factor (VEGF), and a decrease in the pro-inflammatory cytokine IL7. IL13 and IL7 expression tends to reflect a slightly less pro--inflammatory environment after the treatment. VEGF expression was the most affected by immunotherapy with a 5-fold increase. This might be the result of (i) increased soluble Aβ species following plaque solubilisation [25] leading to neurotoxicity and angiogenesis via a VEGF-mediated mechanism [7], or (ii) reflected the vascular side effects reported after immunotherapy, known as amyloid-related imaging abnormalities (ARIA) including microhaemorrhages and oedema [5, 43]. Interestingly, both VEGF and IL8 are involved in angiogenesis [27]. It is worth noting that, from the four markers that differed between AD and controls, IL13 and VEGF were also significantly increased with respect to controls, while IL7 was decreased with respect to control, emphasizing that Aβ-immunotherapy, even though inducing a slightly more anti-inflammatory environment, did not restore the normal state.

## Conclusion

In conclusion, our study supports the experimental observation that microglial motility is impaired in AD, as evidenced by the changes observed in the interactions of the motility-related proteins with each other and with Aβ. As part of the AD pathogenic process, microglia move from baseline to directed-motility, involving all investigated proteins (Iba1, CFL1, CORO1A and P2RY12). Aβ immunotherapy seemed to result in increased microglial baseline motility (suggested by increased Iba1 and P2RY12 expression, which are considered “homeostatic” microglial markers), potentially due to decreased Aβ and/or tau, but without restoration of the physiological status of microglia.

## Supplementary information

**Additional file 1:** **Table S1.** Demographic, clinical and *post-mortem* characteristics of the control group. **Table S2.** Demographic, clinical and *post-mortem* characteristics of the Alzheimer’s disease group. **Table S3.** Demographic, clinical and *post-mortem* characteristics of the immunised Alzheimer’s disease group.

## Data Availability

The data used and/or analysed during the current study are available from the corresponding author on reasonable request.

## Abbreviations

AD: Alzheimer’s disease
ADP: Adenosine diphosphate
ARIA: Amyloid-related imaging abnormalities
ATP: Adenosine triphosphate
Aβ: Amyloid-β
BCA: Bicinchoninic acid assay
CFL1: Cofilin
CORO1A: Coronin 1A
GSK: Glycogen Synthase Kinase
iAD: immunised AD
Iba1: Ionized calcium-binding adaptor molecule 1
ICAM: Intercellular adhesion molecule
IFN: Interferon
IL: Interleukin
IQR: Interquartile range
MSD: MesoScale Discovery
P2RY12: Purinergic receptor P2Y12
ptau: Hyperphosphorylated tau
ROI: Region of interest
TGF: Transforming growth factor
TNF: Tumour necrosis factor
UDP: Uridine diphosphate
UTP: Uridine triphosphate
VEGF: Vascular endothelial growth factor
