# Supplementary material for: Microglial motility in Alzheimer’s disease and after Aβ42 immunotherapy: a human post-mortem study
Source: Acta Neuropathol Commun. 2019 Nov 8;7:174. doi: 10.1186/s40478-019-0828-x (PMC6842157; doi:10.1186/s40478-019-0828-x)
Supplement: Supplementary file 1 — Additional file 1: Table S1. Demographic, clinical and post-mortem characteristics of the control group. Table S2. Demographic, clinical and post-mortem characteristics of the Alzheimer’s disease group. Table S3. Demographic, clinical and post-mortem characteristics of the immunised Alzheimer’s disease group. [file 40478_2019_828_MOESM1_ESM.docx]

**Additional file 1: Table S1 – Demographic, clinical and *post-mortem* characteristics of the control group.**

| **MRC ID** | **Case ID** | **Neuropath diagnosis** | **Gender** | **Age of death** | **Braak stage** | **ABC scores** | **APOE genotype** | **PMD (hours)** |
| --- | --- | --- | --- | --- | --- | --- | --- | --- |
| BBN_8684 | 50 | Control | M | 71 | 1 | B1 | 3.3 | 25 |
| BBN_8691 | 57 | Control | F | 82 | 3 | B2 | 2.3 | 35 |
| BBN_8706 | 72 | Control | M | 72 | 1 | B1 | 3.3 | 42 |
| BBN_8708 | 74 | Control | M | 90 | 2 | B1 | 2.3 | 45 |
| BBN_8709 | 75 | Control | M | 83 | 2 | B1 | 3.3 | 86 |
| BBN_8728 | 98 | Control | F | 88 | 2 | B1 | 3.3 | 62 |
| BBN_8732 | 103 | Control | F | 76 | 2 | B1 | 3.3 | 106 |
| BBN_8735 | 106 | Control | F | 88 | 0 | B0 | 3.3 | 72 |
| BBN_8739 | 110 | Control | F | 93 | 2 | B1 | 3.3 | 18 |
| BBN_8751 | 122 | Control | M | 82 | 2 | B1 | 3.3 | 30 |
| BBN_8770 | 141 | Control | F | 89 | 2 | B1 | 3.3 | 15 |
| BBN_8818 | 189 | Control | F | 93 | 1 | B1 | 3.3 | 15 |
| BBN_8835 | 206 | Control | F | 73 | 1 | B1 | 3.3 | 59 |
| BBN_8888 | 259 | Control | M | 89 | 2 | B1 | 3.4 | 91 |
| BBN_8898 | 269 | Control | F | 83 | 2 | B1 | 3.3 | 24 |
| BBN_8923 | 295 | Control | M | 82 | 2 | B1 | 3.3 | 3 |
| BBN_8964 | 336 | Control | F | 82 | 2 | B1 | 4.4 | 37 |
| BBN_9028 | 402 | Control | M | 76 | 2 | B1 | 3.3 | 23 |
| BBN_9292 | 714 | Control | M | 73 | 3 | A0, B2, C1 | 3.3 | 35 |
| BBN_9329 | 751 | Control | M | 80 | 0 | B0, C0 | 3.3 | 46 |
| BBN_9340 | 762 | Control | F | 94 | 2 | A0, B1, C0 | 2.3 | 21 |
| BBN_9344 | 766 | Control | M | 92 | 2 | A1, B1, C1 | 3.4 | 34 |
| BBN_4205 | 781 | Control | M | 87 | 2 | A0, B1, C0 | 3.3 | 24 |
| BBN_9354 | 786 | Control | M | 85 | 2 | A1, B1, C1 | 3.3 | 31 |
| BBN_9359 | 803 | Control | M | 77 | 1 | B1, C1 | 3.3 | 42 |
| BBN_4229 | 818 | Control | F | 87 | 3 | A0, B2, C0 | 2.3 | 47 |
| BBN_9365 | 826 | Control | F | 86 | 2 | B1, C0 | 3.4 | 32 |
| BBN_9392 | 854 | Control | F | 96 | 2 | B1, C1 | n/k | 64 |
| BBN_9407 | 870 | Control | F | 90 | 2 | B1, C1 | 3.3 | 41 |
| BBN_9408 | 871 | Control | M | 87 | 2 | B1, C1 | 3.3 | 42 |
| BBN_9422 | 887 | Control | F | 74 | 1 | A1, B1, C0 | 3.3 | 39.5 |

AD neuropathological change as ABC score when available from the Brain bank: A=Aβ plaque score, B=neurofibrillary tangle stage, C=neuritic plaque score according to National Institute on Aging-Alzheimer’s Association guidelines [20].

MRC: Medical Research Council; n/k: not known; PMD: *post-mortem* delay

**Table S2 – Demographic, clinical and *post-mortem* characteristics of the Alzheimer’s group.**

| **MRC ID** | **Case ID** | **Neuropath diagnosis** | **Gender** | **Age of death** | **Duration of AD** | **Braak stage** | **ABC scores** | **APOE genotype** | **PMD (hours)** |
| --- | --- | --- | --- | --- | --- | --- | --- | --- | --- |
| BBN_8842 | 213 | AD | F | 81 | 10 | 6 | B3 | 3.4 | 42 |
| BBN_8846 | 217 | AD | F | 84 | 11 | 6 | B3 | 4.4 | 96 |
| BBN_8852 | 223 | AD | F | 71 | 8 | 5 | B3 | 4.4 | 71 |
| BBN_8910 | 282 | AD | M | 71 | 9 | 6 | B3 | 3.3 | 30 |
| BBN_8912 | 284 | AD | F | 82 | 7 | 6 | B3 | 3.4 | 24 |
| BBN_8968 | 340 | AD | F | 89 | 8 | 4 | B2 | 3.3 | 14 |
| BBN_9076 | 451 | AD | F | 84 | 10 | 5 | B3 | 3.4 | 20 |
| BBN_9095 | 470 | AD | F | 80 | 9 | 5 | B3 | 3.4 | 72 |
| BBN_9122 | 498 | AD | F | 83 | 8 | 5 | B3 | 3.3 | 5 |
| BBN_9123 | 499 | AD | F | 73 | 12 | 5 | B3 | 3.3 | 35 |
| BBN_9125 | 501 | AD | F | 84 | 8 | 6 | B3 | 4.4 | 23 |
| BBN_9162 | 538 | AD | M | 66 | 5 | 6 | B3 | 3.3 | 43 |
| BBN_9188 | 567 | AD | F | 68 | 8 | 6 | B3 | 3.4 | 87 |
| BBN_9189 | 568 | AD | F | 78 | 16 | 6 | B3 | 4.4 | 21 |
| BBN_9194 | 573 | AD | F | 89 | 9 | 5 | B3 | 4.4 | 39 |
| BBN_9198 | 577 | AD | F | 79 | 2 | 6 | B3 | 3.4 | 14 |
| BBN_9243 | 665 | AD | F | 88 | 5 | 5 | B3 | 3.4 | 75 |
| BBN_9263 | 685 | AD | M | 74 | 10 | 5 | B3, C2 | 2.3 | 48 |
| BBN_9266 | 688 | AD | M | 79 | 9 | 6 | A3, B3, C3 | 3.3 | 72 |
| BBN_9269 | 691 | AD | M | 83 | 6 | 4 | B2, C2 | 3.4 | 99 |
| BBN_9274 | 696 | AD | M | 77 | 12 | 6 | A3, B3, C2 | 4.4 | 49 |
| BBN_9284 | 706 | AD | F | 76 | 11 | 6 | A3, B3, C2 | 3.4 | 44 |
| BBN_9295 | 717 | AD | M | 86 | 6 | 6 | A3, B3, C3 | 3.3 | 50 |
| BBN_9301 | 723 | AD | F | 84 | 11 | 5 | A3, B3, C3 | 3.4 | 11 |
| BBN_9310 | 732 | AD | F | 85 | 16 | 5 | A3, B3, C3 | 3.4 | 102 |
| BBN_9315 | 737 | AD | F | 67 | 5 | 6 | A3, B3, C3 | 4.4 | 24 |
| BBN_9320 | 742 | AD | F | 87 | 15 | 6 | A3, B3, C3 | 3.3 | 28 |
| BBN_9323 | 745 | AD | F | 84 | 15 | 6 | A3, B3, C3 | 2.3 | 21 |
| BBN_9336 | 758 | AD | M | 86 | 6 | 5 | A3, B3, C2 | 3.3 | 60 |
| BBN_9341 | 763 | AD | F | 80 | 9 | 5 | A3, B3, C3 | 4.4 | 51 |
| BBN_9346 | 768 | AD | F | 85 | 11 | 6 | A3, B3, C3 | 3.3 | 64 |
| BBN_4200 | 773 | AD | M | 69 | 7 | 6 | A3, B3, C3 | 3.3 | 72 |
| BBN_4213 | 792 | AD | M | 88 | 9 | 5 | B3, C2 | 3.4 | 5 |
| BBN_4215 | 794 | AD | F | 80 | 7 | 4 | B2, C2 | 3.4 | 26 |
| BBN_4219 | 802 | AD | F | 70 | 5 | 5 | A3, B3, C3 | 3.4 | 31 |
| BBN_4228 | 816 | AD | M | 84 | 12 | 5 | A3, B3, C3 | n/k | 13 |
| BBN_4233 | 822 | AD | M | 77 | 14 | 6 | A3, B3, C3 | n/k | 59 |
| BBN_9366 | 827 | AD | M | 81 | 20 | 5 | A3, B3, C3 | n/k | 9 |
| BBN_9367 | 828 | AD | M | 77 | 10 | 6 | A3, B3, C3 | 4.4 | 19 |
| BBN_9371 | 833 | AD | F | 73 | 7 | 5 | A3, B3, C3 | 3.3 | 51 |
| BBN_9377 | 839 | AD | M | 75 | 10 | 5 | A3, B3, C3 | 3.3 | 40 |
| BBN_9394 | 856 | AD | M | 81 | 10 | 4 | A3, B2, C2 | 3.4 | 32 |
| BBN_9401 | 864 | AD | F | 87 | 12 | 6 | A3, B3, C3 | 3.3 | 45 |
| BBN_9420 | 885 | AD | F | 89 | 7 | 6 | A3, B3, C3 | 3.4 | 7 |

AD neuropathological change as ABC score when available from the Brain bank: A=Aβ plaque score, B=neurofibrillary tangle stage, C=neuritic plaque score according to National Institute on Aging-Alzheimer’s Association guidelines [20].

AD: Alzheimer’s disease; MRC: Medical Research Council; n/k: not known; PMD: *post-mortem* delay

**Table S3 – Demographic, clinical and *post-mortem* characteristics of the immunised Alzheimer’s group.**

| **Case ID** | **Neuropath diagnosis** | **Gender** | **Age of death** | **Duration of AD** | **Braak stage** | **ABC scores** | **APOE genotype** | **PMD (hours)** |
| --- | --- | --- | --- | --- | --- | --- | --- | --- |
| 1 | iAD | F | 74 | 6 | 6 | A3, B3, C3 | n/k | 30 |
| 2 | iAD | M | 83 | 11 | 6 | A3, B2, C2 | n/k | 6 |
| 3 | iAD | M | 63 | 6 | 5 | A3, B3, C3 | n/k | 10 |
| 4 | iAD | F | 71 | 10 | 6 | A3, B3, C3 | n/k | 22 |
| 6 | iAD | M | 81 | 7 | 6 | A3, B3, C3 | 3.4 | 6 |
| 7 | iAD | M | 82 | 6 | 4 | A1, B3, C0 | 3.4 | 16 |
| 8 | iAD | M | 63 | 10 | 5 | A1, B3, C1 | 3.4 | 6 |
| 9 | iAD | M | 81 | 11 | 5 | A3, B3, C1 | 4.4 | n/k |
| 10 | iAD | F | 88 | 11 | 5 | A3, B3, C3 | 3.3 | 17 |
| 11 | iAD | M | 88 | 12 | 5 | A3, B3, C2 | 3.4 | 8 |
| 16 | iAD | F | 89 | 15 | 6 | A3, B3, C3 | 3.4 | n/k |
| 17 | iAD | F | 86 | 13 | 6 | A3, B3, C0 | 4.4 | 7 |
| 19 | iAD | F | 75 | 19 | 6 | A3, B3, C3 | n/k | 42 |
| 20 | iAD | M | 82 | 17 | 6 | A3, B2, C2 | n/k | 15 |
| 21 | iAD | F | 87 | 18 | 5 | A3, B3, C3 | 3.4 | 96 |
| 22 | iAD | M | 74 | 18 | 6 | A3, B3, C3 | 4.4 | n/k |

AD neuropathological change as ABC score: A=Aβ plaque score, B=neurofibrillary tangle stage, C=neuritic plaque score according to National Institute on Aging-Alzheimer’s Association guidelines [20, 33].

iAD: immunised Alzheimer’s disease; MRC: Medical Research Council; n/k: not known; PMD: *post-mortem* delay
